# Involvement of focal adhesion kinase in cellular invasion of head and neck squamous cell carcinomas via regulation of MMP-2 expression

**DOI:** 10.1038/sj.bjc.6604286

**Published:** 2008-03-18

**Authors:** M Canel, P Secades, M Garzón-Arango, E Allonca, C Suarez, A Serrels, M C Frame, V Brunton, M-D Chiara

**Affiliations:** 1 Servicio de Otorrinolaringología, Hospital Universitario Central de Asturias; 2 Instituto Universitario de Oncologia del Principado de Asturias, Universidad de Oviedo, Oviedo, Spain; 3 Beatson Institute for Cancer Research, Cancer Research UK Beatson Laboratories, Glasgow, UK

**Keywords:** FAK, head and neck cancer, squamous cell carcinoma, FRNK, invasion, MMP-2

## Abstract

Focal adhesion kinase (FAK) is considered intimately involved in cancer progression. Our previous research has demonstrated that overexpression of FAK is an early and frequent event in squamous cell carcinomas of the supraglottic larynx, and it is associated with the presence of metastases in cervical lymph nodes. The purpose of this study was to examine the functional role of FAK in the progression of head and neck squamous cell carcinomas (HNSCC). To this end, expression of FAK-related nonkinase (FRNK) or small interfering RNA (siRNA) against FAK was used to disrupt the FAK-induced signal transduction pathways in the HNSCC-derived SCC40 and SCC38 cell lines. Similar phenotypic effects were observed with the two methodological approaches in both cell lines. Decreased cell attachment, motility and invasion were induced by FRNK and FAK siRNA, whereas cell proliferation was not impaired. In addition, increased cell invasion was observed upon FAK overexpression in SCC cells. FRNK expression resulted in a downregulation of MMP-2 and MMP-9 expression. Interestingly, MMP-2 overexpression in FRNK-expressing cells rescued FRNK inhibition of cell invasion. This is the first demonstration of a direct rescue of impaired cell invasion by the re-expression of MMP-2 in a tumour cell type with decreased expression of functional FAK. Collectively, these data reported here support the conclusion that FAK enhances invasion of HNSCC by promoting both increased cell motility and MMP-2 production, thus providing new insights into possible therapeutic intervention strategies.

Head and neck squamous cell carcinoma (HNSCC) is one of the most common types of human cancer, comprising ∼50% of all malignancies in some developing nations. HNSCC is associated with severe disease- and treatment-related morbidity and has a 5-year survival rate of ∼50%. This survival rate has remained largely unchanged in the past three decades (Sankaranarayanan *et al*, 1998; Gonzalez-Botas and Vazquez Barro, 2006). A major determinant of the lethal progression of HNSCC is the spreading of the malignant cells to regional lymph nodes which represents a major prognostic indicator (Forastiere *et al*, 2001). Thus, attempts to identify the genes involved in metastasis are pivotal for the early prediction of HNSCC behaviour and development of novel molecular therapies. However, the identities of molecular alterations that endow cancer cells with these metastatic functions are largely unknown.

The process of metastasis consists of sequential and selective steps including proliferation, motility, invasion, loss of cell–cell and cell–matrix adhesion, and remodelling of the extracellular matrix. A key factor involved in the control of cell–extracellular matrix interactions is focal adhesion kinase (FAK), an intracellular tyrosine kinase protein that is localised to cellular focal contact sites (Schaller *et al*, 1992). Initially, phosphorylation of FAK occurs on its major autophosphorylation site, Tyr^397^. Phosphorylation of this tyrosine initiates a cascade of signal transduction events that result in the phosphorylation of subsequent tyrosine residues, including Tyr^576^, Tyr^577^, Tyr^861^ and Tyr^925^ which render the molecule a fully active kinase (Schlaepfer *et al*, 1999). It is well-established that FAK plays a crucial role in mediating signal transduction pathways initiated either at the sites of cell attachment or at growth factor receptors (Sieg *et al*, 2000). Its activation leads to a number of cell biological processes, including cell attachment, migration, invasion, proliferation and survival which are crucial for cancer development and metastasis (Gabarra-Niecko *et al*, 2003; McLean *et al*, 2005). A large number of reports describe an enhanced expression of FAK protein in a variety of human cancers, including sarcomas, astrocytomas and carcinomas of the breast, colon, thyroid, prostate, oral cavity, liver, stomach and ovary (see (McLean *et al*, 2005) and references herein). Furthermore, FAK overexpression has been correlated with the invasive potential of a tumour and poor patient prognosis (Cance *et al*, 2000; Miyazaki *et al*, 2003; Recher *et al*, 2004; Sood *et al*, 2004). In a study of 106 matched pairs of larynx carcinomas and corresponding normal tissue, using immunohistochemistry, we have previously shown a significantly increased expression of FAK at the protein level in both the tumour tissues and the corresponding metastasis (Canel *et al*, 2006). Strikingly, we also found that the FAK expression levels of the primary metastatic tumors were maintained in their corresponding lymph node metastases, and FAK protein overexpression in primary tumors correlates with nodal metastasis. These data suggest that the deregulation of FAK in HNSCC may have an active role in the development of distant metastasis to lymph nodes.

In the present report, we address whether FAK expression or activity in two HNSCC-derived cell lines (SCC40 and SCC38) contributes to important aspects of HNSCC tumorigenesis such as growth, cell motility or invasion. The expression of FAK-related nonkinase (FRNK) or small interfering RNA against FAK (siRNA) were used to disrupt the FAK-mediated signal transduction pathways in SCC cells. Exogenous expression of FRNK has been widely used as a dominant-negative mutant to inhibit FAK signalling (Schlaepfer *et al*, 2004). FRNK comprises the C-terminal region of FAK and promotes FAK tyrosine dephosphorylation most likely by the competitive displacement of FAK from integrin-containing focal contacts. The two methodological approaches yielded similar conclusions. We show that inhibition of FAK-signalling in SCC40 and SCC38 cells induced decreased cell attachment, motility and invasion. Furthermore, inhibition of FAK activity also led to decreased MMP-2 gene expression that was associated with the selective loss of the invasive cell phenotype. These data support the conclusion that FAK enhances *in vitro* cell invasion activity, at least in part, by promoting both increased cell motility and MMP-2 production.

## MATERIALS AND METHODS

### Cell culture

The established human HNSCC-derived cell lines SCC-40 and SCC38 were kindly provided by Dr R Grenman (Department of Otolaryngology, University Central Hospital, Turku, Finland). Cells were grown as previously described (Canel *et al*, 2006).

### Transient transfections, generation of FRNK-expression construct and stable SCC40 and SCC38 cell lines

Transient transfections were performed in SCC cells using Fugene 6 (Roche Applied Science, Indianapolis, IN, USA) and following manufacturer's instructions.

The full-length cDNA coding for FRNK was cloned into the *Bam*HI/*Sal*I restriction sites of the retroviral vector pWZL. Phoenix**-**Ampho packaging cells were transfected with the FRNK-expression plasmid or empty vector using Fugene (Roche) in antibiotic-free media. The virus-containing media were collected 48 h posttransfection and immediately used to infect SCC40 and SCC38 cells. Cells were infected at a density of 1 × 10^6^ cells ml^−1^ for 48 h in the presence of 4 *μ*g ml^−1^ of polybrene. The infection procedure was repeated after 24 h. Forty-eight hours after the second infection, 350 *μ*g ml^−1^ of hygromycin was added to the culture medium for selection of infected cells. After 10 days of selection, pooled populations of cells were grown in culture medium with 25 *μ*g ml^−1^ of antibiotic. The hygromycin selective pressure was removed 24 h before experimental procedures. FRNK expression in transfected pool was monitored by western blot analysis and real time PCR.

Stable populations of FRNK-SCC40 cells were stably transfected with pcDNA3-human MMP-2 plasmid. FRNK-SCC40 and -SCC38 cells were also stably transfected with either pCEP-Puro-MMP-2 or pCEP-Puro-mutMMP-2. mutMMP-2 is a catalytically inactive form of MMP-2 described in (Hauck *et al*, 2002). Selection was performed with 350 *μ*g ml^−1^ of G418 (for cells transfected with pcDNA3-MMP-2 plasmid) or 180 *μ*g ml^−1^ of puromycin (for cells transfected with pCEP-Puro-MMP-2 or pCEP-Puro-mutMMP-2 plasmids) for 10 days. Stable pooled populations of FRNK-MMP-2 cells were maintained in culture using 20 *μ*g ml^−1^ of G418 or puromycin.

### siRNA treatment

siRNA duplex oligonucleotides were purchased from Dharmacon Research (Lafayette, CO, USA). The targeted sequences for FAK siRNAs were: GAUAGUGGACAGUCACAAA, CCAGUUUACUGAAGAUAAG, UUUCUUCUAUCAACAGGUG. siCONTROL Nontargeting pool (Dharmacon) were used as control siRNA. SCC40 and SCC38 cells were transfected with 35 pmol ml^−1^ siRNAs using Lipofectamine 2000. Protein analyses revealed a substantial inhibition of FAK expression 48–72 h after transfection. The transfected cells were used for subsequent experiments within that interval of time.

### Confocal immunofluorescence microscopy

SCC-40 cells were transiently transfected with pEGFP-FRNK plasmid and grown on BD Falcon culture slides. Cells were then fixed in TBS with 3.7% paraformaldehyde and permeabilised by incubation with TBS containing 0.5% Triton X-100 and 1% bovine serum albumin for 15 min. After blocking with 5% fetal bovine serum in TBS for 1 h, SCC40 cells transiently transfected with pEGFP-FRNK were incubated with phalloidin-TRITC at 1 : 100 dilution for 40 min for actin organisation. pWZL- and FRNK-SCC40 cells were incubated with rabbit anti-vinculin (Sigma) and mouse anti-FAK (Becton-Dickinson) at 1 : 100 dilutions for 16 h at 4°C, and then washed in phosphate-buffered saline (PBS) and incubated with CY™2-conjugated anti-mouse and CY™3-conjugated anti-rabbit IgG. After washings in TBS/0.025% Tween-80, cells were examined using a confocal microscope (Leica TCS-SP2-AOBS).

### MTS-based cell proliferation assay

MTS assays were performed using CellTiter 96 Cell NonRadioactive Proliferation Assay following the protocol recommended by the manufacturer (Promega, Madison, WI, USA). Briefly, 1000 cells were seeded in each well of 96-well plates, and allowed to grow for 48, 72 or 96 h. MTS assay was performed at each time point.

### Cell cycle and apoptosis analyses

For cell cycle analysis, we used the Vindelov staining method as described (Vindelov *et al*, 1983). Cells (5 × 10^5^) were stained with propidium iodide (100 *μ*g ml^−1^) and RNase A (100 *μ*g ml^−1^) for 10 min. Cell cycle distribution was acquired with a Cytomics FC500 flow cytometer (Beckman Coulter). For apoptosis analyses, cells (5 × 10^5^) were harvested, washed with PBS, and resuspended in dual staining solution containing 0.5 *μ*g ml^−1^ of propidium iodide and 0.2 *μ*g ml^−1^ of FITC-Annexin V (FITC-Annexin V/PI protocol; Sigma). Cells were then incubated for 10 min at room temperature in the dark and analysed by flow cytometry within 1 h. All analyses were performed in triplicate.

### Cell attachment

Cells were harvested by trypsinisation and labelled in culture medium with 5 *μ*M fluorescent dye (calcein AM, Molecular Probes) at 37°C for 1 h. After washing, cells were resuspended in DMEM, plated (3 × 10^5^ cells per well) on a 96-well tissue culture dish coated with fibronectin, type I collagen, matrigel or poly-HEMA (Sigma), and incubated for 40 min at 37°C. Following incubation, the unattached cells were rinsed away with PBS. The fluorescence signal from the adherent cells was measured by using a fluorescence plate reader (FLEXstation™, Molecular Devices) at an excitation wavelength of 494 nm and an emission wavelength of 517 nm. All analyses were performed in triplicate.

### Wound healing assay

Cells were grown to confluence in 35-mm tissue culture dishes. Cell monolayers were wounded using a micropipette tip, and floating cells were removed by extensive washing with DMEM. Photographs of the wounded area were taken immediately after making the scratch (0 h time point) and after 20 h to measure the migration rate of cells into the wounded area. At least 15 different fields were randomly chosen across the wound length.

### Time-lapse video recording of cell motility

Cells were seeded in 6-well plates coated with fibronectin, collagen or matrigel. The cell linear movements were monitored using an Axiovert 200 M Zeiss microscope with a × 20 objective. Images were captured at 15-min intervals for 12 h from five different fields in each well. About 100 individual cells per cell line were analysed using Tracking Analysis software (Kinetic Imaging). All analyses were performed in triplicate.

### Matrigel invasion assays

*In vitro* invasion assays were performed by using a 24-well invasion chamber coated with Matrigel (Becton Dickinson). Cells were trypsinised, washed with PBS, suspended in DMEM containing 5% bovine serum albumin (BSA), and plated in the invasion chamber (3 × 10^4^ cells per well). The lower chambers were filled with DMEM containing 5% BSA with either 10% (for SCC40 cells) or 2.5% fetal bovine serum (for SCC38 cells). After 24 h, the cells remaining in the upper chamber were removed by scraping, whereas the cells that invaded through Matrigel were fixed and stained by using 0.5% crystal violet in methanol. All invading cells were counted by microscopic visualisation. In the case of SCC38 cells transiently transfected with pcDNA3-MMP-2, invasion occurred as group of cells and, therefore, quantification was performed by extracting the crystal violet dye with dimethylsulphoxide followed by spectrophotometry at 590 nm. All analyses were performed in triplicate.

### Gelatin zymography

Cells (80% confluent) were incubated in serum-free DMEM at 37°C for at least 18 h. The media were then collected and clarified by centrifugation to remove cells and debris. Samples were loaded under nonreducing conditions onto 7.5% SDS–polyacrylamide gel containing 0.2% (w/v) gelatin. Following electrophoresis, the gels were washed with 2.5% Triton X-100 to remove SDS followed by incubation in a developing buffer (20 mM Tris, pH 7.4, 5 mM CaCl_2_) overnight at 37°C. Gels were stained with Coomassie Brilliant Blue R-250 and destained. Gelatinase activity was visualised as clear bands against the blue-stained gelatin background. HT-1080 fibrosarcoma cells-conditioned medium, containing high expression levels of MMP-2 and MMP-9 proteins, was used as positive control and migration standards. Three individual experiments were conducted with independent protein samples.

### Western blot analyses

Protein extracts were obtained from SCC40 and SCC38 cells at 80–90% confluence as previously described (Canel *et al*, 2006). Equal amounts of proteins were fractionated on SDS–PAGE and transferred to PVDF membranes. Membranes were probed with anti-FAK clone 4.47 (Upstate Biotechnology, Lake Placid, NY, USA); anti-FRNK, anti-paxillin (Becton Dickinson Transduction Laboratories, Erembodegem, Belgium), anti-pY397 FAK, anti-pY861 FAK (Biosource, Camarillo, CA, USA); anti-pY118 paxillin (Cell Signaling Technology Inc.); anti-pY925 FAK or anti-*α*-tubulin (Sigma-Aldrich, St Louis, MO, USA) at 1 : 1.000 dilutions. Bound antibodies were detected using enhanced chemiluminescence reagent (Amersham Pharmacia Biotech) according to the protocol of the manufacturer.

### Quantitative real-time RT–PCR

Total RNA was isolated from cells as previously described (Del Toro *et al*, 2003). First-strand cDNA was synthesised from 2 *μ*g of total RNA using the Superscript first-strand synthesis system for reverse transcriptase (Invitrogen, Carlsbad, CA, USA) with random primers according to the manufacturer's directions. Real-time PCR was done in an ABI Prism 7500 Sequence Detection System (Applied Biosystems, Foster City, CA, USA) using SYBR Green PCR Master mix (Applied Biosystems) and the thermocycler conditions recommended by the manufacturer. Each sample was analysed for cyclophilin A to normalise for RNA input amounts and to perform relative quantification. Primers were designed using the computer program Primer Express (Applied Biosystems). The primers used were as follows: FAK, forward, 5′-CTTCGGACAGCGTGAGAGAGA-3′ and reverse, 5′-GACGCATTGTTAAGGCTTCTTGA-3′; MMP-2, forward, 5′-TGCTGGAGACAAATTCTGGAGATA-3′ and reverse, 5′-GGATCCATTTTCTTCTTCACCTCAT-3′; MMP-9, forward, 5′-ACCTCGAACTTTGACAGCGAC-3′ and reverse, 5′-CAAACTGTATCCTTGGTCCGG-3′; cyclophilin, forward, 5′-CATCTGCACTGCCAAGACTGA-3′ and reverse, 5′-TTGCCAAACACCACATGCTT-3′.

## RESULTS

### Inhibition of FAK-mediated signalling by expression of FRNK or siRNA against FAK

We have previously reported that FAK is overexpressed in tumour tissue samples and cell lines derived from HNSCC (Canel *et al*, 2006). Here, to analyse the functional significance of FAK in HNSCC progression, we disrupted the FAK-mediated signal transduction pathway by expression of the FAK COOH-terminal domain (FAK-related nonkinase, FRNK) or small interfering RNA (siRNA) against FAK in the HNSCC-derived SCC40 and SCC38 cell lines. Among the HNSCC-derived cell lines used in our previous report (Canel *et al*, 2006), we have selected SCC40 and SCC38 cells as systems to study the phenotypic effects of inhibiting FAK, because they express high levels of FAK and the predominant location of activated FAK (pTyr^397^-FAK) is at focal adhesion sites.

**Figure 1:**
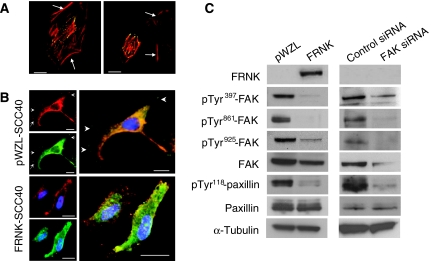
Inhibition of FAK-mediated signalling by expression of FRNK or siRNA against FAK in HNSCC-derived SCC40 cells. (**A**) Confocal microscopy images of SCC40 cells transiently transfected with pEGFP-FRNK and stained with phalloidin-TRITC to visualise actin organisation (GFP-FRNK, green fluorescence; phalloidin-TRITC, red fluorescence). Images show transfected cells as well as nontransfected cells (marked with arrows), which served as internal controls. As shown, FRNK is localised to focal contacts, docking sites of actin cytoskeleton to the extracellular matrix. Scale bars, 10 *μ*m. (**B**) Confocal microscopy images of SCC40 cells stably transfected with pWZL vector alone or pWZL-FRNK (FAK, green fluorescence; vinculin, red fluorescence). Images show that FAK colocalise with vinculin at focal contact sites (marked with arrows) in pWZL-SCC40 cells whereas is mainly detected in the cytoplasm in FRNK-SCC40 cells. Scale bars, 20 *μ*m (pWZL-SCC40) and 15 *μ*M (FRNK-SCC40). (**C**) Western blot analysis showing expression of FRNK, pTyr^397^-FAK, pTyr^861^-FAK, pTyr^925^-FAK, total FAK, pTyr^118^-paxillin and total paxillin in SCC40 cells stably transfected with pWZL-FRNK (FRNK) or treated with FAK-siRNA. Cells infected with empty pWZL vector (pWZL) or transfected with control siRNA served as the control. The membranes were stripped and reprobed with anti-*α*-tubulin antibody to assure even loading of proteins in each lane.

Expression of FRNK has been widely used as a dominant-negative inhibitor of FAK function. To determine whether FRNK may compete with FAK at focal adhesion site in a HNSCC-derived cell line, GFP-tagged FRNK was transiently expressed in SCC40 cells. Confocal microscopy images showed that FRNK was localised to focal contacts, docking sites of actin cytoskeleton to the extracellular matrix (Figure 1A). Therefore, FRNK is likely to function as an inhibitor of FAK signalling in SCC40 cells.

We next established stable pool populations of FRNK- or empty vector-expressing SCC40 cells (FRNK and pWZL, respectively). The subcellular localisation of endogenous FAK in transformed cells was evaluated. As shown in Figure 1B, FAK mainly co-localised with vinculin, a focal adhesion component, in pWZL cells whereas it was also found in the cytoplasm in FRNK-SCC40 cells. The retroviral-mediated expression of FRNK in SCC40 cells did not substantially affect the overall levels of FAK but resulted in the attenuation of FAK phosphorylation at Tyr^397^, a site whose phosphorylation is associated with FAK-kinase activation. FRNK also impaired the phosphorylation of FAK at Tyr^861^ and Tyr^925^, two phospho-tyrosines that act as docking sites for protein interactions crucial for FAK signalling function (Figure 1C). FRNK expression did not affect the expression of paxillin, a FAK-associated focal adhesion component. However, the phosphorylation of paxillin at Tyr^118^ decreased in FRNK cells as compared with pWZL cells, consistent with a model of FAK as an upstream modulator of focal adhesion components phosphorylation (Ruest *et al*, 2001). Stable pool populations of SCC38 cells were also obtained and shown to behave as described for SCC40 cells (data not shown). Thus, FRNK expression provides a useful tool to dissect the cellular mechanism by which FAK promotes tumour cell phenotypes in HNSCC.

Nevertheless, given that expression of FRNK may cause effects not related to the inhibition of FAK activity, we also examined the effects of double-stranded siRNA oligonucleotides against FAK in SCC40 and SCC38 cells. As shown in Figure 1C, FAK expression was dramatically suppressed after 72 h of FAK siRNA transfection in SCC40 cells, whereas control siRNA showed no effect. Similar inhibition was also found in SCC8 cells treated with FAK siRNA (data not shown). As expected, the levels of pTyr^397^-, pTyr^861^-, and pTyr^925^-FAK were also markedly decreased. Suppression of FAK expression by siRNA was also accompanied by decreased levels of pTyr^118^-paxillin.

Taken together, the data showed that the two methodological approaches, expression of FRNK and siRNA against FAK, inhibited FAK-mediated signalling pathways in HNSCC-derived cells, and were suitable tools to address the question as to whether FAK is involved in HNSCC progression.

### Inhibition of FAK expression or activity does not induce changes in cell proliferation

**Figure 2:**
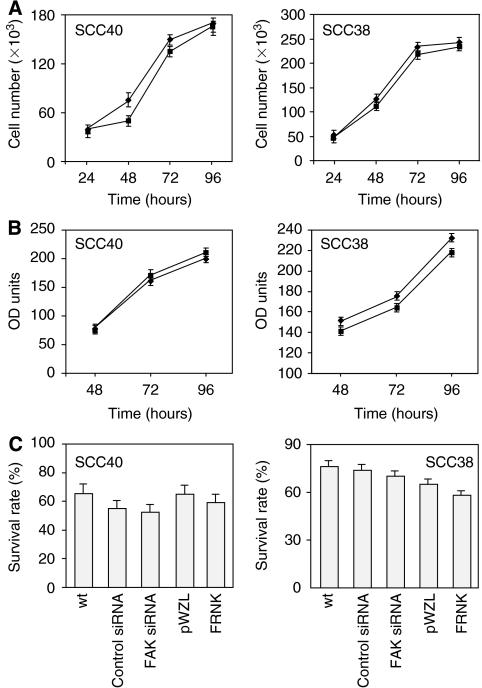
FAK inhibition does not affect cell proliferation or survival in SCC cells. (**A**) Cell proliferation of pWZL-(♦) and FRNK-SCC cells (▪). Cells (50 × 10^3^) were plated and 24, 48, 72, or 96 h later the number of cells was measured by direct counting of trypan blue-excluding cells using a hemacytometer. Values are mean±s.d. from a representative experiment performed in triplicate in a series of three. (**B**) MTS assays were performed with pWZL-(♦) and FRNK-SCC cells (▪) growth for 48, 72 and 96 h. Values are mean±s.d. from a representative experiment performed in triplicate in a series of three. (**C**) Survival rate of FRNK- or pWZL-SCC cells and SCC cells treated with FAK- or control-siRNA. The fraction of viable cells was measured by using the FITC-Annexin V apoptosis detection kit. Values are mean of average±s.d. from three independent experiments done in triplicate.

Previous studies have shown that inhibition of FAK function by FRNK results in decreased cell proliferation in nonneoplastic cells (reviewed in (Craven *et al*, 2003; Parsons, 2003)). In addition, FAK has been directly implicated in the promotion of tumour growth and cell cycle progression in some tumour cells (Aguirre Ghiso, 2002; Hecker *et al*, 2002, 2004; van Nimwegen *et al*, 2005). To investigate the possible role of FAK on cell proliferation of HNSCC cells, MTS assays and cell counting were performed in SCC40 and SCC38 cells expressing FRNK, siRNA against FAK, and in their corresponding control cells. As shown in Figure 2, inhibition of FAK activity by FRNK expression did not significantly affect the cell growth rates. Similar data were obtained with siRNA-treated cells (data not shown). Accordingly, the number of cells in each phase of the cell cycle was similar in FRNK cells as compared with pWZL cells, and in SCC cells transfected with FAK siRNA *vs* control siRNA (data not shown). The apoptotic index was also evaluated showing that both FRNK and FAK-siRNA expression did not significantly affect cell viability in SCC cells (Figure 2C). These data show that in the tumour background examined here FAK is not important for cell proliferation.

### Inhibition of FAK expression or activity impairs cell attachment

**Figure 3:**
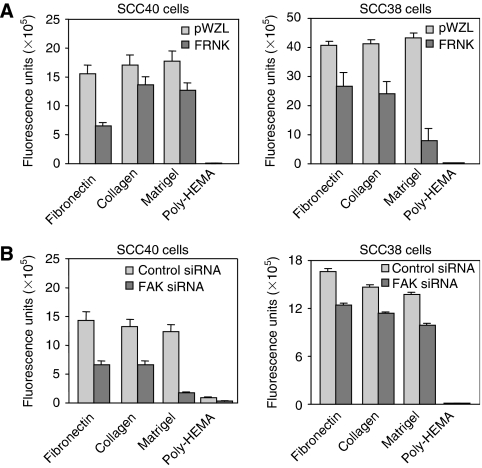
Inhibition of FAK expression or activity impaired cell attachment in SCC40 and SCC38 cells. Cell adhesion on different substrates was determined as described in Material and Methods in SCC40 and SCC38 cells stably transfected with pWZL-FRNK (**A**) and cells treated with FAK-siRNA (**B**) and compared with that measured in their corresponding control cells (pWZL and control siRNA). Values are mean of average±s.d. from four independent experiments done in triplicate.

As cell attachment to the extracellular matrix may contribute to cancer metastasis, we investigated the effect of inhibition of the FAK-mediated signal transduction on early cell adhesion of SCC40 and SCC38 cells. The experiments were conducted using three different components of the extracellular matrix as substrates. Stable FRNK expression induced inhibition of cell attachment in both SCC40 and SCC38 cells independently of the component of the extracellular matrix used as a substrate (Figure 3A). The percentage of inhibition in SCC40 cells ranged from about 25% (when the cell adhesion assay was performed with collagen or matrigel) to ∼60% (when fibronectin was used as a substrate). In SCC38 cells, the highest rate of inhibition (∼80%) was detected when the cell adhesion assay was performed with matrigel. In the same manner, cell adhesion to all substrates tested decreased in SCC40 cells transfected with FAK siRNA as compared with cells transfected with control siRNA (Figure 3B). The highest rate of inhibition of cell attachment (∼85% reduction *vs* control cells) was observed in the presence of a reconstituted matrix gel whereas adhesion to other substrates was inhibited about 50%. SCC38 cells treated with FAK siRNA displayed about 25–30% inhibition of cell adhesion to all substrates as compared with control cells. As expected, cell adhesion to poly-HEMA, a polymer that acts as an inhibitor of cell adhesion (Folkman and Moscona, 1978), was negligible with all cells tested.

### Inhibition of FAK-mediated signalling attenuates cell migration and motility

**Figure 4:**
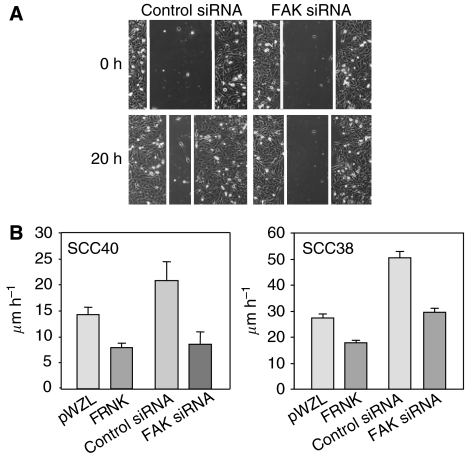
Inhibition of FAK-mediated signalling pathway attenuates cell migration in SCC40 and SCC38 cells. Wound healing assays were performed in FRNK- and pWZL-expressing SCC40 and SCC38 cells, and cells treated with FAK- or control-siRNA. (**A**) Representative images captured with a × 10 objective at the time of wounding (0 h) or 20 h after wounding (20 h) in SCC40 cells transfected with control- or FAK-siRNA. (**B**) Rate of front migration of cell monolayers analysed by time-lapse video microscopy. At least 15 different fields were randomly chosen across the wound length. Values are mean of average±s.d. from three independent experiments.

To examine the effect of inhibition of FAK expression/activity on cell migration, confluent monolayers of SCC40 or SCC38 cells expressing FRNK, FAK siRNA or their respective controls were artificially wounded by scraping them with a pipette tip and filmed by time-lapse microscopy as they moved into the wound. As shown in Figure 4B, the dominant negative FRNK decreased the migratory potential of both SCC40 and SCC38 cells by ∼44 and ∼35%, respectively as compared with pWZL cells. Similarly, knock down of FAK expression by siRNA resulted in a 59% (SCC40 cells) or 41% (SCC38 cells) decrease in cell migration as compared with cells transfected with nonspecific siRNA (Figure 4B). Figure 4A shows a representative image of wound healing assay performed in SCC40 cells transfected with control or FAK siRNAs.

**Figure 5:**
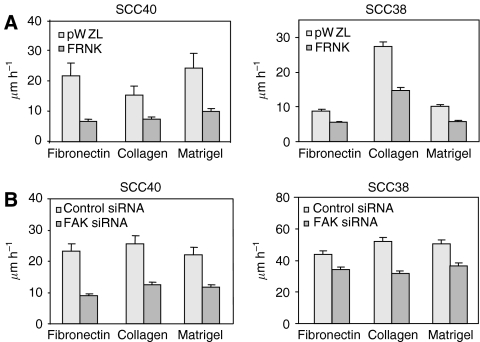
Inhibition of FAK-mediated signalling inhibits cell motility in SCC40 and SCC38 cells. FRNK- or pWZL-SCC40 and SCC38 cells (**A**) and cells treated with FAK- or control-siRNA (**B**) were plated and images taken every 15 min over a 12-h period. Quantification of linear cell motility was determined by tracing the single cells using the cell-tracking software (Kenetic Imaging Ltd). About 100 individual cells per cell line were analysed. Values are mean of average±s.d. from three independent experiments done in triplicate.

Random linear cell motility was also assessed in cells carrying inhibition of FAK-signalling. Cells expressing FRNK, FAK siRNA or their respective control cells were plated on three different substrates; fibronectin, collagen and matrigel, at low densities in the presence of serum, and their random motility were monitored by time-lapse microscopy. FRNK cells displayed reduced random cell motility in the three substrates tested, varying from 79% of inhibition on fibronectin, to 52% on collagen, and 59% on matrigel for SCC40 cells (Figure 5A). The rate of inhibition was about 50% in FRNK-SCC38 cells when the assay was performed with any of the three substrates. Inhibition of FAK expression with siRNA in SCC40 and SCC38 cells caused similar inhibitory effect on cell motility (Figure 5B). In this case, the rates of inhibition were 47–62 and 20–40% for SCC40 and SCC38 cells, respectively. Time-lapse video microscopy also revealed that FRNK cells while anchored to the substrate still exhibited broad membrane protrusions that projected in multiple directions, a phenomenon known as membrane ruffling (see Supplementary information). In the case of cells transfected with FAK siRNA, besides refractory cell motility, we detected that they maintained a more elongated morphology than control cells as they were either unable to release their trailing edges or they simultaneously moved in opposite directions (see Supplementary material). These phenotypes could be due in part to an enhanced stability of focal contact structures (Ilic *et al*, 1995).

### Inhibition of FAK-mediated signalling pathway impairs cell invasion and MMP-2 expression

**Figure 6:**
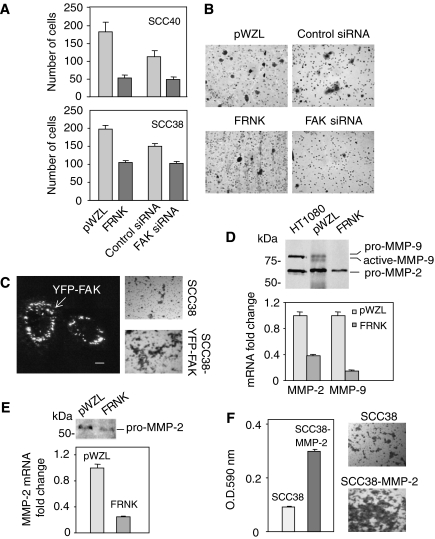
Inhibition of FAK-mediated signalling decreases cellular invasion through Matrigel and MMP-2 expression in SCC cells. (**A** and **B**) FRNK- or pWZL-SCC cells and SCC cells treated with FAK- or control-siRNA were seeded in serum-free media in the upper chamber of Matrigel transwells. The lower chamber was loaded with regular media supplemented with 10% fetal bovine serum and 5% BSA. After 24 h at 37°C in 5% CO_2_, the top filter was scraped, and invading cells were fixed and stained. (**A**) All invading cells were counted under × 10 magnification. Values are mean of average±s.d. from a representative experiment in a series of three done in triplicate. (**B**) Representative images from three separate experiments performed in SCC40 cells. (**C**) SCC38 cells were transiently transfected with either pYFP-FAK or vector alone. Picture on the left is a representative confocal microscope image showing that YFP-FAK fusion protein localises at focal sites. Scale bars, 10 *μ*m. Matrigel invasion assays were performed 24 h after transfection. Representative images from three separate matrigel invasion experiments performed in SCC38 cells transfected with either vector alone or pYFP-FAK are shown on the right. (**D** and **E**), FRNK- or pWZL-SCC40 (**D**) and FRNK- or pWZL-SCC38 (**E**) cells were cultured in serum-free media. After 24 h, conditioned media and cells were harvested for gelatinase zymography and MMPs mRNA quantification, respectively. HT-1080 fibrosarcoma cells conditioned medium was used as positive control for zymography and migration standards. MMP-2 and MMP-9 transcripts were quantified using quantitative RT–PCR. The mean of relative expression to cyclophilin A housekeeping gene of at least three independent experiments is shown. (**F**) Matrigel invasion assay was performed in SCC38 cells 24 h after transfection with either pcDNA3-MMP-2 or vector alone. Cell staining with crystal violet and subsequent extraction with dimethylsulphoxide followed by spectrophotometry at 590 nm was performed for quantification of invading cells. All analyses were performed in triplicate. Representative images from three separate experiments are shown.

SCC40 and SCC38 cells expressing FRNK and FAK siRNA were analysed for their invasive potential through a ∼1-mm Matrigel barrier compared with cells transfected with empty vector or nonspecific siRNA. The data revealed that invasion was inhibited with both, FRNK and FAK siRNA expression in SCC40 cells, showing a 70 and 60% decrease in invasiveness, respectively (Figure 6A and B). Similarly, inhibition of FAK activity or expression in SCC38 cells resulted in 50 and 35% decrease of cell invasion in FRNK- and FAK siRNA-expressing cells, respectively (Figure 6A). We next determined whether FAK overexpression increases the invasion potential of SCC cells. As shown in Figure 6C, exogenously expressed FAK was localised in focal adhesion sites in SCC38 cells transiently transfected with a yellow-FAK (YFP-FAK) fusion protein. Matrigel invasion assays showed that the invasion ability of SCC38 cells was significantly enhanced (about twofold increase as compared with control cells) after transient transfection with YFP-FAK (Figure 6C).

Because elevated expression, activation, and/or secretion of matrix metalloproteinases (MMPs) in transformed cells can promote an invasive phenotype (Deryugina and Quigley, 2006), experiments were performed to determine whether enhanced MMPs secretion and/or activity were associated with FAK-mediated cell invasion. Conditioned media were collected from FRNK or pWZL-SCC40 cells and subjected to gelatin zymography. HT-1080 fibrosarcoma cells conditioned medium was used as positive control for gelatin zymography. As shown in Figure 6D, analysis of pWZL-conditioned media revealed the presence of a major MMP protein activity that comigrated with pro-MMP-2 (72 kDa, gelatinase A). Low levels of both pro-MMP-9 (92 kDa, gelatinase B) and activated-MMP-9 were also detected. Real time RT–PCR analyses confirmed the reduced expression levels of MMP-9 as compared with MMP-2 in SCC40 cells (MMP-2 mRNA levels were about 30-fold higher than MMP-9 levels; data not shown). Gelatinase zymography comparisons between FRNK and pWZL cells revealed a strong inhibition of both MMP-2 and MMP-9 protein expression in FRNK *vs* pWZL cells. Quantitative real time RT–PCR was then performed to determine whether MMPs protein inhibitions were also detected at the mRNA level. The data revealed that, in accordance with the zymography analysis, MMP-2 and MMP-9 mRNA levels were, respectively, 62 and 85% lower in FRNK cells as compared with pWZL cells (Figure 6D). These results are consistent with recent studies showing a positive role for FAK in mediating MMP-2 and MMP-9 secretion in other cell types (Sein *et al*, 2000; Hauck *et al*, 2002; Zhang *et al*, 2002; Hsia *et al*, 2003; Hu *et al*, 2006; Mitra *et al*, 2006).

We further determined whether lower levels of MMPs expression and secretion were also present in FRNK-SCC38 cells *vs* pWZL-SCC38 cells. In these SCC cells, MMP-2 but not MMP-9 protein was detected by gelatin zymography. MMP-9 mRNA was also undetectable by real time RT–PCR experiments. Similar to SCC40 cells, FRNK expression in SCC38 cells resulted in inhibition of MMP-2 expression detected at both, protein and mRNA levels (Figure 6E). Expression of FAK siRNA in SCC38 cells also resulted in a significant 3-fold reduction in MMP-2 mRNA levels (data not shown). For unknown reasons, this last result could not be reproduced in SCC40 cells.

We then determined whether MMP-2 could be involved in the invasive potential of SCC cells by analysing the *in vitro* invasion activity of SCC38 cells transiently transfected with either pcDNA3-MMP-2 or empty vector. Matrigel invasion assays (Figure 6F) revealed that a significantly greater number of MMP-2 transfected cells invaded the Matrigel compared with the vector control cells. FAK mRNA levels were not modified by MMP-2 overexpression (data not shown). Taken together, these data show that MMP-2 is a potential mediator of the FAK activity on cell invasion in SCC cells.

### MMP-2 overexpression rescues FRNK inhibition of cell invasion

**Figure 7:**
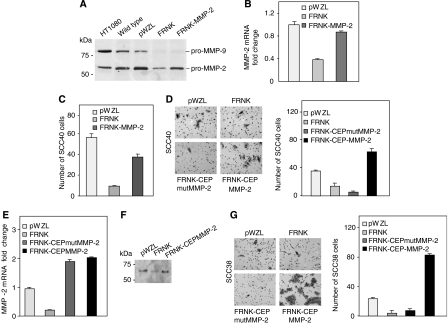
MMP-2 overexpression rescues FRNK inhibition of cell invasion in SCC40 (**A**–**D**) and SCC38 (**E**–**G**) cells. Gelatinase zymography (**A**), MMP-2 mRNA quantification (**B**) and *in vitro* invasive assays (**C**) were performed in FRNK-, pWZL-, and FRNK-MMP-2-SCC40 cells as indicated in Figure 6. (**D**) *In vitro* invasive assays performed in pWZL-, FRNK-, FRNK-CEP-MMP-2- and FRNK-CEPmutMMP-2-SCC40 cells. All invading cells were counted under × 10 magnification. Representative images from three separate experiments are shown. MMP-2 mRNA quantification (**E**), gelatinase zymography (**F**) and matrigel invasion assays (**G**) were performed in pWZL-, FRNK-, FRNK-CEP-MMP-2- and FRNK-CEPmutMMP-2-SCC38 cells as indicated above. Representative images of matrigel invasion assays in SCC38 cells are shown in (**G**). Experiments were repeated at least three times.

To determine whether the inhibitory effect of FRNK in cell invasion is mediated by MMP-2 reduced expression, human MMP-2 was stably overexpressed in FRNK-expressing SCC40 and SCC38 cells. In SCC40 cells, pooled populations of FRNK-MMP-2 overexpressing cells showed increased levels of both MMP-2 protein, as detected by zymography analysis, and MMP-2 mRNA levels, as detected by real time RT–PCR analyses (Figure 7A and B). No changes in MMP-9 expression were detected in cells overexpressing MMP-2. Western blot analysis showed that FAK protein levels were not modified by MMP-2 overexpression in FRNK SCC cells (data not shown). Overexpression of MMP-2 in FRNK cells did not induce changes in cell attachment but slightly increased (1.5 fold increase *vs* FRNK cells) cell migration (data not shown). Analysis of cell invasion through matrigel in FRNK cells overexpressing MMP-2 showed a fourfold increase in cell invasion as compared with FRNK cells (Figure 7C). To rule out that the observed MMP-2 rescue of invasion was a nonspecific effect, a pCEP-Puro plasmid containing either wild type MMP-2 cDNA or cDNA encoding for a catalitically inactive mutant MMP-2 protein was stably transfected in FRNK-SCC40 cells. Transfected cells showing equal levels of exogenous MMP-2 or mutMMP-2 (FRNK-CEP-MMP-2 and FRNK-CEPmutMMP-2 cells in Figure 7D) were selected for analysis of cell invasion. Re-expression of MMP-2, but not mutant MMP-2, in FRNK-cells notably increased cell invasion to levels even higher than those detected in pWZL-SCC38 cells (Figure 7D).

Stably re-expression of MMP-2 or mutant MMP-2 was also performed in FRNK-SCC38 cells. FRNK-CEP-MMP-2 pooled clones showed increased levels of MMP-2 protein as compared with FRNK-SCC38 cells (Figure 7F). Accordingly, increased MMP-2 mRNA levels were detected in FRNK-CEP-MMP-2 and FRNK-CEPmutFRNK cells as compared with pWZL- and FRNK-SCC38 cells (Figure 7E). No changes in FAK protein expression levels were detected in cells overexpressing MMP-2 (data not shown). We then analysed cell invasion through matrigel of FRNK-CEP-MMP-2 or FRNK-CEPmutMMP-2 cells and compared with that of pWZL- and FRNK-SCC38 cells. The data showed that re-expression of MMP-2, but not mutant MMP-2, in FRNK-cells notably increased cell invasion to levels even higher than those detected in pWZL-SCC38 cells (Figure 7G). These data support the conclusion that FAK enhances *in vitro* SCC cell invasion activity by promoting MMP-2 secretion.

## DISCUSSION

We have previously reported that FAK is overexpressed in tumour tissue samples and cell lines derived from HNSCC (Canel *et al*, 2006). Importantly, FAK overexpression in these carcinomas was found in all stages of cancer progression, and it was associated with the presence of lymph node metastasis, thus suggesting that the deregulation of FAK may have an active role in the invasion process of this type of tumors. It is well-documented that FAK regulates cellular properties that could mediate its oncogenic activity such as cell adhesion, proliferation, migration, invasion and survival (Mitra and Schlaepfer, 2006; van Nimwegen and van de Water, 2007). However, FAK seems to have different roles in different tumour types contributing to different aspects of malignancy (Hauck *et al*, 2002; Hsia *et al*, 2003; Han *et al*, 2004; Mitra *et al*, 2006). The current study addressed the role of FAK in mediating the processes that promote the invasive phenotype of HNSCC. We inhibited FAK function in HNSCC cells by stable expression of a dominant-negative variant of FAK, FRNK, or transient expression of siRNA against FAK. Collectively, the data suggest that FAK is an important mediator of cell attachment, migration and invasion of HNSCC cells. We also show that FAK enhances *in vitro* SCC40 and SCC38 cell invasion activity, at least in part, by promoting MMP-2 secretion. Notably, these cancer cells were not dependent on FAK for continued proliferation.

We show that inhibition of FAK signalling by expression of FAK or FAK siRNA did not affect cell proliferation or survival in SCC40 and SCC38 cells. Several studies have indicated that FAK may have a direct role in tumour growth and cell survival (Xu *et al*, 1996, 1998; Sonoda *et al*, 1999, 2000; Aguirre Ghiso, 2002; Hecker *et al*, 2002, 2004; van Nimwegen *et al*, 2005). However, there are also published data showing that FRNK expression induces defects on migration and/or invasion without effects on cell survival or proliferation (Han *et al*, 2004; Mitra *et al*, 2006). Indeed, impairment of cell proliferation and survival after FRNK expression in carcinoma cells has been associated with high levels of overexpression (Xu *et al*, 2000; Golubovskaya *et al*, 2002; van Nimwegen *et al*, 2005). Therefore, the contradictory effects on cell growth or survival may be due to the levels of functional FAK in the presence of FRNK or FAK siRNA. Alternatively, cell background differences may explain the resistance to inhibition of these phenotypes.

Our data revealed that FRNK and FAK siRNA expression in SCC40 and SCC38 cells induced decreased cell attachment, motility and invasion. These phenotypes agree with the previous reported data in other tumour cell types (Hauck *et al*, 2002; Schlaepfer *et al*, 2004; Mitra *et al*, 2005). Cellular migration is a complex process that requires the precise cooperation of various signal transduction pathways, which facilitate the regulated assembly and disassembly of focal contacts to coordinate attachment and detachment of the cell from the extracellular matrix. FAK has been found to be a key regulator of cellular migration by initiating many of the signal transduction pathways necessary for the assembly and turnover of focal contacts. Although our *in vitro* data support a role for FAK in the adhesion and migration processes involved in metastasis formation, the exact downstream mediators of these effects remain to be identified. The initial assembly of integrin-associated focal adhesions requires FAK autophosphorylation on Tyr^397^ and complex formation with Src. The FAK-Src signalling complex functions to recruit and phosphorylate various FAK-associated proteins including paxillin (Bellis *et al*, 1995; Schaller *et al*, 1995) which participates in the coordination of integrin-mediated cell motility (Klemke *et al*, 1998; Petit *et al*, 2000). We show here that FRNK and siRNA against FAK inhibited Tyr^397^ phosphorylation of FAK and Tyr^118^ phosphorylation of paxillin in HNSCC-derived cells, which is accompanied by decreased cell motility. Nevertheless, impairment of cell motility in FRNK-expressing cells is apparently accompanied by increased focal contact formation in membrane ruffles thus suggesting that FRNK could still recruit other signalling or effectors proteins required for assembly of focal adhesion sites, and that decreased motility is due to impaired focal adhesion turnover rather than to weakened adhesion assembly. A role of FAK in cell movement acting preferentially in focal adhesion turnover rather than in initial formation has been previously suggested (Schlaepfer *et al*, 2004), and phosphorylation of both FAK and paxillin contributes to turnover of focal contacts (Turner, 2000; Brunton *et al*, 2005; Hamadi *et al*, 2005). Possibly, the decreased cell motility caused by inhibition of FAK expression or activity in HNSCC-derived cells is due to a decreased FAK phosphorylation and FAK-induced paxillin phosphorylation and, consequently, impaired focal adhesion turnover. Nevertheless, the mechanism of action of FAK is complex and probably involves multiple downstream signalling substrates.

Inhibition of FAK-mediated signalling pathway in SCC40 and SCC38 cells also impairs cell invasion through matrigel. In addition, transient overexpression of FAK in SCC cells increases cell invasion. Several reports have shown that FAK can regulate cell invasion not only by regulation of cell migration but also by the modulation of matrix metalloproteinases that degrade extracellular matrix barriers (Hsia *et al*, 2003; Mon *et al*, 2006b). We found that MMP-2 and MMP-9 gene expression were greatly reduced by FRNK expression in SCC40 cells. SCC38 cells express negligible levels of MMP-9, as detected by both mRNA quantification and gelatinase activity. Nevertheless, as observed in FRNK-SCC40 cells, FRNK-SCC38 cells showed reduced levels of MMP-2 gene expression and secretion. We also show here that MMP-2 is able to promote cell invasion in SCC cells. Previous studies have supported a role for FAK in MMP-2 secretion in response to environmental factors (Sein *et al*, 2000; Zhang *et al*, 2002; Mon *et al*, 2006b). Our studies show that FAK is implicated in MMP-2 gene expression leading to increased MMP-2 secretion in SCC cells. Consistent with our findings, previous reports in v-Src transformed fibroblasts and glioma cells (Hauck *et al*, 2002; Hu *et al*, 2006) supported a role for FAK in promoting increased MMP-2 gene expression. This FAK signalling pathway also seems to operate in noncancerous cells (Segarra *et al*, 2005). There have been reports linking other extracellular proteases, such as MMP-9 (Mon *et al*, 2006a), MMP-1 (Zeng *et al*, 2006) and uPA (Mitra *et al*, 2006) with FAK signalling in other tumour cell types, but until now MMP-2 has not been reported to be involved. Therefore, distinct downstream signalling pathways are active in a tumour cell type-dependent manner. Evaluation of FAK-specific signalling in different tumour types is therefore helpful for development of the therapeutic strategies for cancers with high FAK expression levels. In the present report, in addition to showing a reduced expression of MMP-2 after FAK inhibition, we show that overexpression of MMP-2 in FRNK-expressing HNSCC-derived cell lines blocked FRNK-induced inhibition of cell invasion. The reversion of the FRNK blockage of cell invasion was complete in both cell lines, which reached levels of invasion 2- to 3-fold greater than those observed in pWZL cells. Notably, a catalytically inactive mutant form of MMP-2 did not reverse the effect of FRNK on cell invasion in SCC38 and SCC40 cells. These data demonstrate that the FRNK effects on cell invasion were mediated by the reduced production of MMP-2. With the exception of transformed fibroblasts (Hauck *et al*, 2002), re-expression of MMP-2 in cancer cells with impaired function of FAK and subsequent rescue of *in vitro* cell invasion defects has not been previously studied. The extent to which MMP-2 contributes to FAK-mediated cell invasion in other cancer cells remains unknown. Here we report, for the first time in cancer cells, direct evidence that MMP-2 is an important player of FAK-mediated cell invasion in HNSCC-derived cells.

The biochemical mechanism involved in FAK-mediated regulation of MMP-2 expression in SCC cells is presently unknown. Previous reports have shown a functional link between FAK-ERK1/2-JNK and MMP-2 expression in tumour and noncancerous cells (Hauck *et al*, 2002; Segarra *et al*, 2005; Hu *et al*, 2006). In SCC cells, the constitutive or EGF-induced levels of activated ERK1/2 and JNK were not reduced by FRNK expression in comparison with pWZL-SCC cells (data not shown). These preliminary data suggest that the mediators of the FAK-MMP-2 pathway in HNSCC may differ from those previously reported in other cancer cells. The regulation of MMP-2 gene expression involves the convergence of multiple trans-activators that seems to operate in a cell type-specific manner (Yan and Boyd, 2007). The precise mechanism of FAK-mediated regulation of MMP-2 in HNSCC is being further explored.

Previous studies have determined that both MMP-2 and MMP-9 are associated with lymph node metastasis (Xie *et al*, 2004) and poor outcome (Katayama *et al*, 2004) in HNSCC. In addition, microarray gene expression studies on whole HNSCC tumour samples have identified the overexpression of several MMPs including MMP-2 (Nagata *et al*, 2003; Chung *et al*, 2004). Lastly, MMP-2 is among the recently identified predictive genes that show a positive correlation with lymph node metastasis (Roepman *et al*, 2006). In addition to the facts that FAK overexpression in primary tumours of patients with HNSCC is associated with lymph node metastasis and that there is an almost perfect correlation between the expression levels of FAK found in the primary tumour and in the corresponding metastasis, observations from these studies support the importance of FAK as a mediator of metastatic HNSCC cancer progression via the regulation of MMP-2 expression. Collectively, the data reported here support the conclusion that FAK enhances *in vitro* HNSCC cell invasion activity, at least in part, by promoting both increased cell motility and MMP-2 secretion; thus, these data provide new insights into possible therapeutic intervention strategies for HNSCC.

## Supplementary Material

Supplementary Movie1

Supplementary Movie2

Supplementary Movie3

Supplementary Movie4
